# Aging-associated DNA methylation of LEF1 modulates inflammation and neurodegenerative pathways

**DOI:** 10.3389/fimmu.2025.1656442

**Published:** 2025-08-21

**Authors:** Mengke Chen, Lujie Zhou, Yidan Gao, Yao Zhong, Cheng Chen, Zhicheng Wang, Xiaoning Wang, Rong Xia

**Affiliations:** ^1^ Department of Blood Transfusion, Huashan Hospital, Fudan University, Shanghai, China; ^2^ Department of Hematology, Huashan Hospital, Fudan University, Shanghai, China; ^3^ Shanghai Institute of Infectious Disease and Biosecurity, Shanghai Medical College, Fudan University, Shanghai, China; ^4^ Department of Blood Transfusion, The First Hospital of Jilin University, Changchun, China

**Keywords:** aging, DNA methylation, inflammation, LEF1, microglia

## Abstract

**Background:**

Aging is accompanied by profound changes in immune regulation and epigenetic landscapes, yet the molecular drivers underlying these alterations are not fully understood.

**Methods:**

Transcriptional profiles of peripheral blood samples from young and elderly individuals, together with aging-associated methylation probe data, were used to identify aging biomarkers. Transcriptomics and chromatin immunoprecipitation sequencing (ChIP-Seq) were conducted to explore potential regulatory mechanisms. Functional validation was performed via knockdown in immune and microglial cell lines, assessing inflammatory responses and reactive oxygen species (ROS) levels. Finally, an independent cohort was recruited to validate expression and promoter methylation, with ChIP-seq and the assay for transposase-accessible chromatin with sequencing (ATAC-seq) analyses supporting epigenetic repression as the underlying mechanism.

**Results:**

LEF1 expression was significantly downregulated in elderly individuals, accompanied by increased promoter methylation, indicating age-related epigenetic repression. Integrated multi-omics analysis linked LEF1 to immune inflammation and neurodegenerative pathways. LEF1 knockdown enhanced inflammatory responses and ROS production in vitro. ChIP-seq and ATAC-seq data supported epigenetic repression as a mechanism for age-related LEF1 silencing.

**Conclusions:**

Age-related epigenetic repression of LEF1 contributes to immune-inflammatory activation and may underlie neurodegenerative processes.

## Introduction

Aging is a complex, progressive, and irreversible biological process characterized by a gradual decline in physiological functions over time (1–3). In recent years, with advances in society and healthcare, the aging population has been rapidly increasing. Age is the major risk factor for numerous prevalent human diseases, including cancer, diabetes, cardiovascular disorders, and neurodegenerative diseases (4–6). Consequently, the prevention and treatment of age-related diseases have become a central focus of biomedical research. Most conventional clinical biomarkers do not comprehensively reflect the biological mechanisms of aging, though recent advances—such as DNA methylation clocks—have improved our ability to quantify biological aging more accurately (7). Identifying and validating molecular targets for interventions aimed at extending human lifespan remains a significant challenge.

The mechanisms underlying aging are diverse and encompass twelve recognized hallmarks, including epigenetic alterations, loss of proteostasis, and chronic inflammation (2, 4). Senescent cells develop a senescence-associated secretory phenotype, characterized by the secretion of interleukins, cytokines, chemokines, growth factors, and extracellular matrix proteases, which collectively contribute to aging-related biological functions in both health and disease (8–11). Aging is also commonly accompanied by a state of chronic low-grade inflammation, which is considered a key contributor to neuroinflammation and the development of multiple age-associated diseases (12–14).

Studies suggest that epigenetic alterations are a primary driver of aging in mammals, and restoration of epigenomic integrity may reverse signs of aging (15, 16). Age-associated epigenetic changes encompass a range of modifications, including altered DNA methylation patterns, aberrant post-translational modifications of histones, dysregulated chromatin remodeling, and dysfunctional non-coding RNAs (2, 17, 18). Among these, DNA methylation has received particular attention over the past decade in aging and age-related disease research, as site-specific methylation changes during aging have been shown to predict future health outcomes and lifespan (19–21). “Epigenetic clocks,” which measure changes in hundreds of specific CpG sites, can accurately estimate chronological age across multiple species, including humans, and currently represent the most robust biomarkers for predicting human mortality (7, 22, 23). In gene promoters, hypomethylated CpG sites are generally associated with actively and constitutively expressed genes, whereas hypermethylated CpG sites are typically linked to gene silencing or low expression levels (24).

In recent years, the rapid advancement of high-throughput sequencing technologies has enabled the widespread application of multi-omics data at the molecular level, including transcriptomic, epigenomic, and chromatin accessibility data for systematic analysis of gene expression and epigenetic regulation. These approaches have significantly enhanced our understanding of complex biological processes and disease mechanisms.

In this study, we identified aging-associated blood biomarkers by analyzing transcriptomic data from peripheral blood samples of young and elder healthy volunteers, integrated with DNA methylation information. Among these, we focused on LEF1, which harbors aging-associated methylation probes in its promoter region. Our findings suggest that LEF1 may regulate pathways related to immune inflammation and neurodegenerative diseases. Knockdown of LEF1 in immune cells and microglial cells led to a marked increase in inflammatory responses. In addition, LEF1 deficiency resulted in elevated levels of ROS, indicating enhanced oxidative stress. Importantly, we confirmed that during aging, increased methylation in the LEF1 promoter region contributes to its transcriptional repression through epigenetic silencing. Taken together, LEF1 emerges as a key factor in the aging process and may serve as a potential therapeutic target for aging-related diseases.

## Materials and methods

### Transcriptome data acquisition

The transcriptomic data GSE58137, GSE65219, GSE129917, and GSE62420 were obtained from the publicly available Gene Expression Omnibus (GEO) database (https://www.ncbi.nlm.nih.gov/geo/) maintained by the National Center for Biotechnology Information (NCBI). GSE58137 and GSE65219 include healthy volunteers from different age groups, including both young and elderly individuals, with transcriptomic sequencing performed on their peripheral venous blood samples (25). A summary of demographic characteristics for all included samples is provided in **Supplementary Table S1**. GSE129917 contains expression data from Jurkat cells transfected with LEF1 siRNA to investigate the function of LEF1 (26). GSE62420 provides microglia-specific transcriptomic profiles from mouse brain tissues (27).

### Differential expression analysis

Differential analysis of the datasets was performed using the “limma” package (28), with a significance threshold of *P*-value < 0.05 and |*log_2_Foldchange*| > 1 to identify differentially expressed genes (DEGs) associated with aging or LEF1. The overlapping DEGs associated with aging across different datasets were identified, and a Venn diagram was generated using the “VennDiagram” package (29).

### Immune cell infiltration analysis

To investigate the immune characteristics across different age groups, we used CIBERSORT (30) to analyze the proportions of 22 immune cell subtypes in the largest sample dataset, GSE65219. The infiltration patterns of various immune cell types across samples were visualized using a histogram and a heatmap, generated with the “ggplot2” and “pheatmap” packages.

### Correlation analysis and gene enrichment analyses

We analyzed the relationships between LEF1 and other genes using the R package “Hmisc” to calculate correlation coefficients and *P*-values, applying a significance threshold of *r* > 0.8, *P*-value < 0.05. Visualizations were generated via the “ggplot2” package.

Enrichr (https://maayanlab.cloud/Enrichr/) (31) was used for functional and pathway enrichment analyses. The data were visualized using “ggplot2”.

### Protein–protein interaction network analysis and hub gene identification

PPI network analysis was performed using the online database STRING (version 12.0) (https://string-db.org/) (32). To ensure network reliability, an interaction composite score > 0.4 was set as the minimum threshold for interactions. The PPI network was visualized using Cytoscape software (version 3.9.0) (33). The CytoHubba plugin was employed to calculate the degree and MCC values for each intersection, identifying hub genes. The top 10 ranked genes were selected for further analysis.

### Aging-related methylation probes and epigenetic data acquisition

All methylation probes associated with aging traits and located near CpG islands (including CpG islands and their surrounding regions: N_Shore, S_Shore, N_Shelf, and S_Shelf) were downloaded from the Epigenome-Wide Association Study (EWAS) database (https://ngdc.cncb.ac.cn/ewas/atlas) (34). Epigenetic data, including ChIP-seq and ATAC-seq data, were obtained from the ENCODE database (https://www.encodeproject.org/) (35) and visualized using IGV (version 2.11.2) (36).

### Vector construction

To construct the shRNA expression vector, the plasmid pKLV-U6gRNA (BbsI)-PGKpuro2ABFP (50946, Addgene) was linearized using BbsI (R3059L, NEB, Ipswich, USA) and BamHI (R0136S, NEB), followed by gel purification. Two sets of shLEF1 oligonucleotides were synthesized by Tsingke Biotechnology (Shanghai, China). The sense and antisense strands of shLEF1 were annealed to form complementary double-stranded DNA fragments, which were then ligated into the linearized 50946 plasmid. The ligated plasmid was subsequently transformed into Escherichia coli Stbl3 receptor cells. Colonies containing the shLEF1 plasmid were cultured in LB medium supplemented with 100 μg/mL ampicillin. All recombinant plasmids were verified by sequencing at Tsingke. The shRNA oligonucleotide sequences used are listed in **Table 1**.

**Table 1:** ShRNA oligonucleotide sequences targeting LEF1.

Name	Sequence (5’-3’)
shRNA1-LEF1-FOR	CACCGCGTTGCTGAGTGTACTCTAAACTCGAG TTTAGAGTACACTCAGCAACGTTTTTTTG
shRNA1-LEF1-REV	GATCCAAAAAAACGTTGCTGAGTGTACTCTAAACTCGAG TTTAGAGTACACTCAGCAACGC
shRNA2-LEF1-FOR	CACCGATCCCGAGAACATCAAATAAACTCGAG TTTATTTGATGTTCTCGGGATTTTTTTTG
shRNA2-LEF1-REV	GATCCAAAAAAAATCCCGAGAACATCAAATAAACTCGAG TTTATTTGATGTTCTCGGGATC

### Cell culture

All cell lines were obtained from our laboratory. Jurkat cells were cultured in RPMI-1640 medium supplemented with 10% fetal bovine serum and 1% penicillin/streptomycin. HEK-293T and HMC3 cells were cultured with 10% fetal bovine serum, 90% Dulbecco’s modified Eagle’s medium, and 1% penicillin/streptomycin. All cells were maintained at 37°C in a 5% CO_2_ atmosphere.

### Lentivirus production and cell transduction

Lentivirus carrying the target plasmid was produced by co-transfecting HEK293T cells with the packaging plasmid psPAX2 (12260, Addgene) and the envelope plasmid pMD2.G (12259, Addgene) using Lipofectamine 2000 (Invitrogen). Fresh media were exchanged 6 h after transfection. After 72 hours, the viral supernatant was collected, centrifuged at 1,000g for 10 minutes at 4°C to remove debris, aliquoted, and stored at -80°C.

For stable expression, Jurkat and HMC3 cells were transduced with the lentivirus. After 24 hours, the medium was replaced with fresh culture medium. Transduced cells were then selected with 1 μg/mL puromycin for 72 hours.

### Human samples and PBMC isolation

Healthy individuals were recruited after obtaining informed consent for this study. The inclusion criteria were as follows (1): age-matched participants; (2) availability of blood samples for PBMC isolation; and (3) no history of diabetes or other malignant diseases. The study was approved by the Institutional Review Board and Ethics Committee of Huashan Hospital, Fudan University. Participant demographic information is provided in **Supplementary Table S1**.

PBMCs were freshly isolated from human peripheral blood using Ficoll-Paque (GE Healthcare). After density gradient centrifugation, the PBMC layer was carefully collected and washed twice with PBS. The cell pellet was then resuspended for further experiments.

### RNA extraction and real-time PCR

Total RNA was extracted from human PBMC using Trizol reagent (Thermo Fisher Scientific). Once the RNA was obtained, it was reverse transcribed into cDNA using the cDNA Synthesis Kit (RR036A, TaKaRa) according to the manufacturer’s instructions. Subsequently, amplification and quantification were performed using RT-qPCR on the QuantStudio™ 7 Pro Real-Time PCR System (Applied Biosystems) with Taq Pro Universal SYBR qPCR Master Mix (Vazyme). The relative expression levels were calculated using the 2^–ΔΔCT^ method. Housekeeping gene GAPDH expression was used as an internal reference. The primers used are listed in **Table 2**.

**Table 2:** Real-time qPCR primers used in this study.

Gene	Forward (5’-3’)	Reverse (5’-3’)
LEF1	CTACCCATCCTCACTGTCAGTC	GGATGTTCCTGTTTGACCTGAGG
SOCS1	TTCGCCCTTAGCGTGAAGATGG	TAGTGCTCCAGCAGCTCGAAGA
SOCS3	CATCTCTGTCGGAAGACCGTCA	GCATCGTACTGGTCCAGGAACT
GAPDH	GTCTCCTCTGACTTCAACAGCG	ACCACCCTGTTGCTGTAGCCAA

### Enzyme-linked immunosorbent assay

The culture supernatants from LEF1-knockdown Jurkat and HMC3 cells were collected, and ELISA was performed according to the instructions of the kit. The absorbance value at 450 nm was detected by microplate reader to calculate the expression levels of TNF-α and IL-6. Cytokine concentrations were calculated based on a standard curve. ELISA kits were obtained from Jianglai Biotechnology (Shanghai, China).

### ROS detection analysis

The intracellular ROS generation was evaluated using the EVOS M5000 imaging system (Invitrogen). According to the manufacturer’s instructions, intracellular ROS levels were analyzed using Reactive Oxygen Species Assay Kit (S0033, Beyotime). The cells were treated and rinsed with PBS, then incubated at 37 °C for 20 min in 5% CO_2_ with DCFH-DA (10 µmol/L, 1 mL) in PBS. Finally, after three washes with PBS, fluorescence images of cells under different treatments were captured using a fluorescence microscope.

### Methylation-specific PCR

Genomic DNA was extracted from PBMCs in blood samples using the DNA Isolation Kit (DP304, TIANGEN). The genomic DNA was then treated with a DNA bisulfite conversion kit (DP215, TIANGEN) to convert unmethylated cytosines into uracils. MethPrimer (https://www.methprimer.com/) (37) was used to predict CpG islands in the LEF1 promoter region and design primer sequences. This study used two primers, one specific for methylated DNA and the other for unmethylated DNA. The primer sequences are listed in **Table 3**. The expected PCR products for methylated and unmethylated DNA were 143 bp and 147 bp, respectively. MSP was performed using a methylation-specific PCR kit (EM101, TIANGEN). The PCR products were analyzed by 2% agarose gel electrophoresis.

**Table 3:** Methylation-specific PCR primers targeting LEF1.

Primer type	Forward (5’-3’)	Reverse (5’-3’)
M-143bp	CGAGTTAGGTTGAGAAATTCGA	CTCCGCAATAAAAAACACTACG
U-147bp	GGTGAGTTAGGTTGAGAAATTTGA	AACTCCACAATAAAAAACACTACAAA

### Statistical analysis

All experiments were repeated at least three times. Statistical analyses were performed using R (version 4.4.1) and GraphPad Prism 10. Data are presented as mean ± SEM. Detailed statistical analyses for each experiment can be found in the corresponding figure legends. Unless otherwise specified, statistical comparisons were conducted using an unpaired two-tailed Student’s *t*-test, as indicated in the figure legends. A *P*-value < 0.05 was considered statistically significant.

## Result

### Identification of aging-related blood biomarkers and characteristics of immune aging

**Figure 1:**
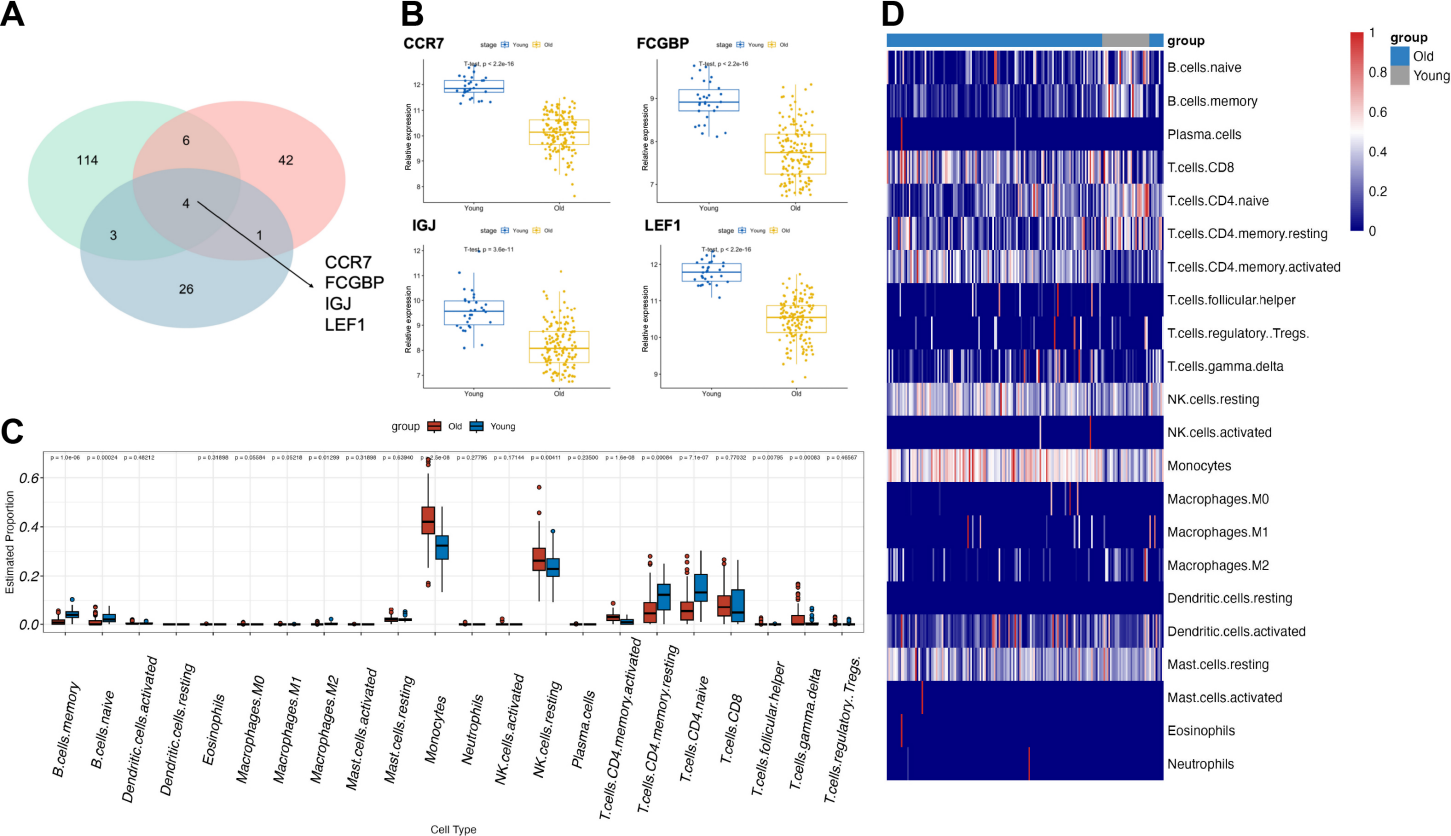
Identification of aging-related blood biomarkers and immune cell population dynamics. **(A)** The intersection of differentially expressed genes from the three datasets identified four aging-related blood biomarkers: CCR7, FCGBP, IGJ, and LEF1. **(B)** The expression levels of the four aging-related blood biomarkers across different age groups. **(C, D)** Changes in immune cell subpopulation proportions in peripheral blood across different age groups based on immune infiltration analysis.

To identify aging-related blood biomarkers, we included peripheral blood data from three cohorts of healthy volunteers (GSE58137_GPL6947/GPL10558 and GSE65219). After grouping the samples by age, we performed differential expression analysis for each dataset and identified four common aging-related blood biomarkers: CCR7, FCGBP, IGJ, and LEF1 (**Figure 1A**). These biomarkers exhibited significant differential expression between elderly and young individuals. Furthermore, in the dataset with the largest sample size (GSE65219), we examined the expression levels of these four biomarkers and found that their expression markedly declined with increasing age (**Figure 1B**). Additionally, we performed immune infiltration analysis to examine the dynamic changes in peripheral blood cell subpopulation proportions across different age groups. We observed that during aging, the proportions of monocytes, activated CD4+ memory T cells, and resting NK cells increased, whereas the proportions of B cells, naïve CD4+ T cells, and resting CD4+ memory T cells decreased. These findings suggest that the immune system may be in a state of chronic inflammation or immunosenescence (**Figures 1C, D**).

In recent years, aging-related research has made unprecedented progress, with epigenetics, particularly DNA methylation, playing a mechanistic role in the aging process as one of the nine hallmarks of aging (7). Therefore, we downloaded all aging-associated methylation probes located near CpG islands (including CpG islands and their surrounding regions: N_Shore, S_Shore, N_Shelf, and S_Shelf) from the EWAS database (**Supplementary Table S2**) (34). Upon annotation, we found that only the promoter region of LEF1-related transcripts contained aging-associated methylation probes. Moreover, previous studies have suggested that LEF1 regulates cellular senescence and aging, and its expression consistently declines with age. Therefore, among the four identified aging-related blood biomarkers, we focused on LEF1 for further investigation.

### LEF1 regulates immune signaling and neurodegenerative disease-associated pathways

**Figure 2:**
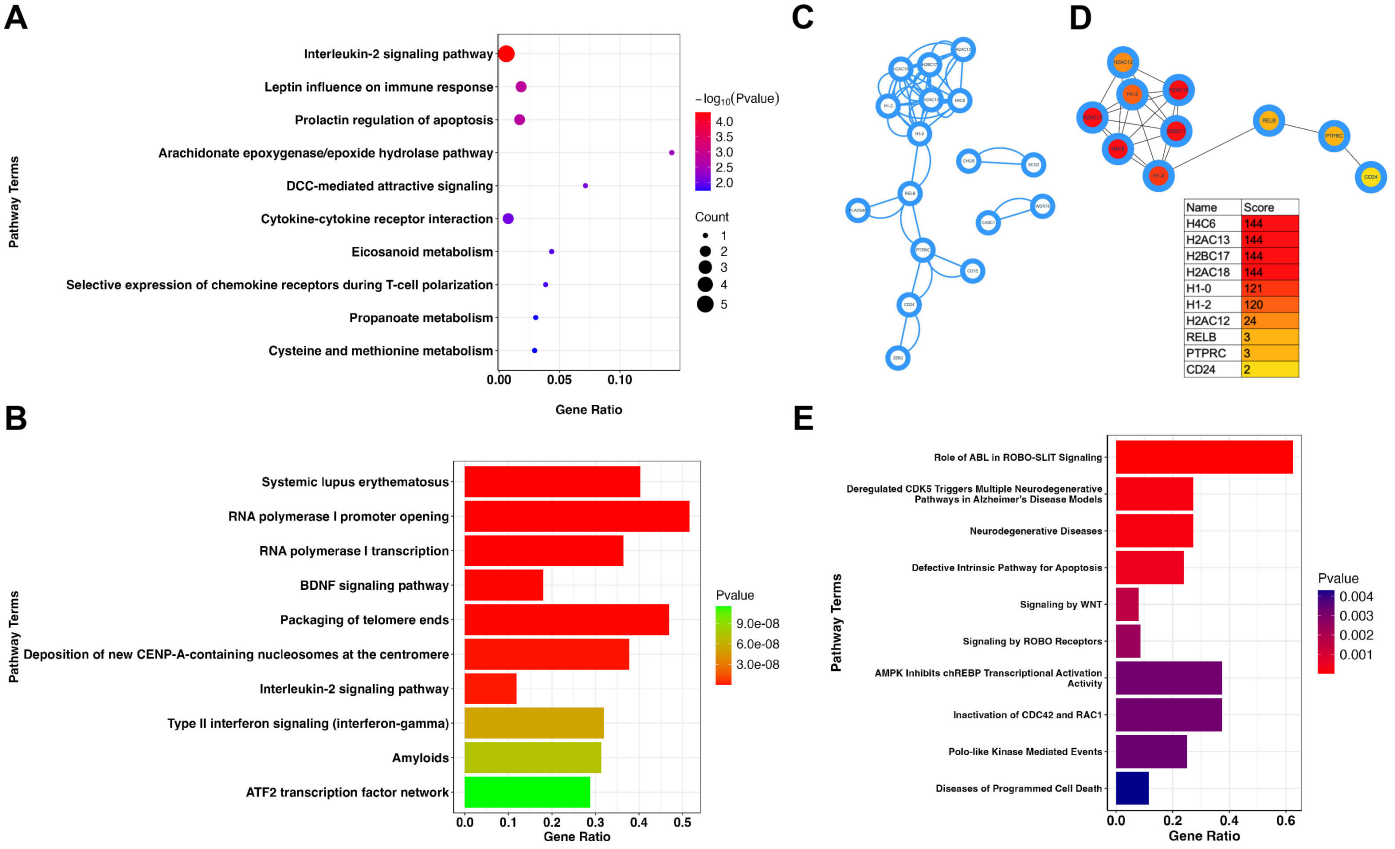
LEF1 regulatory network in immune response and neurodegenerative pathways. **(A)** Enrichment analysis of LEF1 co-expressed genes suggests that LEF1 is associated with the interleukin-2 (IL-2) signaling pathway. **(B)** Enrichment analysis of differentially expressed genes after LEF1 knockdown. **(C)** Protein-protein interaction analysis of differentially expressed genes regulated by LEF1. **(D)** Top 10 hub genes regulated by LEF1. **(E)** ChIP-seq analysis revealed LEF1-binding target genes, and enrichment analysis suggested the association with neurodegenerative diseases.

To further understand the role of LEF1 in the aging process, we first performed a correlation analysis of LEF1 expression in the GSE65219 dataset. We identified co-expressed genes with a correlation coefficient greater than 0.8, as listed in **Supplementary Table S3**. Notably, another aging-related blood biomarker, CCR7, was positively correlated with LEF1 expression (*R* = 0.875, *P* < 0.05). Furthermore, gene enrichment analysis revealed that LEF1 co-expressed genes were primarily associated with the interleukin-2 signaling pathway, which plays a critical role in regulating T-cell proliferation, differentiation, survival, and function within the immune system (**Figure 2A**).

Subsequently, we utilized RNA sequencing data from GEO (GSE129917), which includes Jurkat cells subjected to siRNA targeting LEF1, to further explore the gene network regulated by LEF1 and its potential involvement in aging-related pathways. Through differential gene enrichment analysis (| *logFC* | > 1, *P* < 0.05), our results suggest that LEF1 may influence immune response pathways, including systemic lupus erythematosus, the interleukin-2 signaling pathway, and type II interferon signaling, by regulating associated genes. Additionally, LEF1 may also affect the BDNF signaling pathway and the amyloid pathway, with the BDNF signaling pathway playing a crucial role in neuronal growth, survival, and function, suggesting that LEF1 may be involved in cognitive regulation and neurodegenerative processes (**Figure 2B**). Furthermore, we conducted a PPI analysis of differentially expressed genes using the STRING database and visualized the results in Cytoscape (**Figure 2C**). Using the MCC algorithm in the *cytoHubba* plugin, we identified the top 10 hub genes, which included histone genes (H4C6, H2AC13, H2AC18) as well as immune and signaling-related genes (CD24, PTPRC, RELB) (**Figure 2D**). These findings suggest that LEF1 may not only influence gene expression and chromatin regulation but also participate in immune signaling modulation, tumor-related pathways, and the NF-κB pathway, thereby affecting cellular states and functions. The detailed information on the 10 hub DEGs is presented in **Supplementary Table S4**.

As a nuclear transcription factor, LEF1 specifically binds to cis-regulatory DNA elements (e.g., promoters or enhancers) to regulate target gene expression. Using ChIP-seq data from the ENCODE database, we identified potential LEF1 target genes. Enrichment analysis revealed that LEF1 is associated with genes linked to neurodegenerative diseases, suggesting its regulatory role in neurodegenerative pathways (**Figure 2E**). LEF1-associated target genes related to neurodegenerative diseases, such as EGR1, RHOB, and ID2, exhibit binding peaks (**Supplementary Figures S1A-C**). Following siRNA-mediated LEF1 knockdown, their expression levels were reduced (**Supplementary Figure S1D**). Downregulation of these genes affects key processes, including synaptic plasticity, neuroinflammation, axon regeneration, and neuronal survival, which are implicated in neurodegenerative diseases and brain injury repair (38–40).

### LEF1 deficiency disrupts immune homeostasis and enhances inflammatory responses

**Figure 3:**
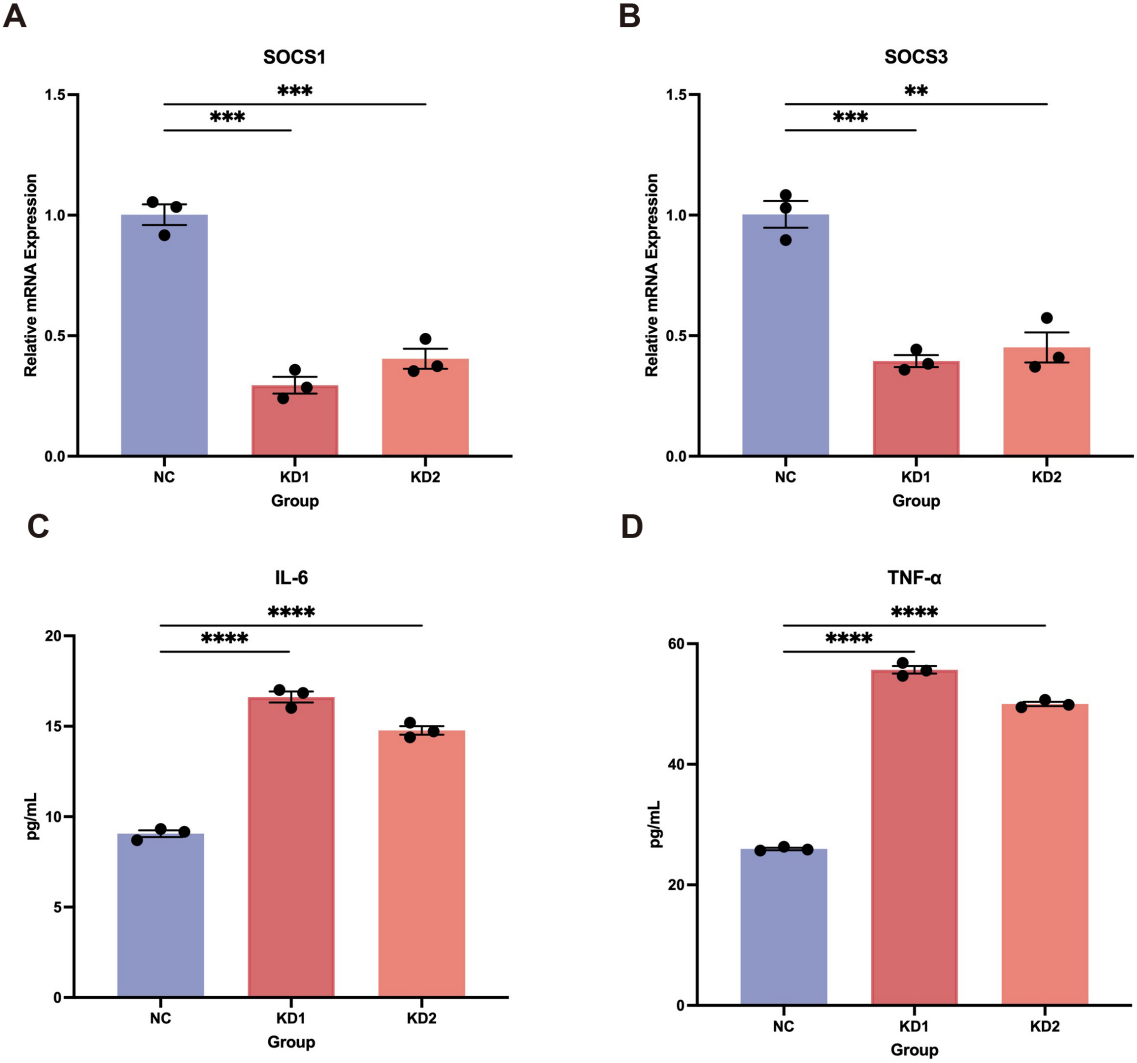
Pro-inflammatory effects induced by LEF1 knockdown. **(A, B)** RT-qPCR confirmed the downregulation of SOCS1 and SOCS3 following LEF1 knockdown. **(C, D)** ELISA confirmed increased levels of IL-6 and TNF-α in the cell supernatant following LEF1 knockdown, indicating elevated inflammation. Data are presented as the mean ± SEM of three independent samples, and the *P* values were analyzed using a two-tailed unpaired *t* test. **P<0.01, ***P<0.001, ****P<0.0001.

Using the DICE and ImmuNexUT databases, we found that LEF1 exhibits the highest expression levels in T cells (**Supplementary Figures S2A, B**). Therefore, we further investigated the function of LEF1 by establishing an LEF1-knockdown human T cell line (Jurkat cells). RT-qPCR experiments confirmed LEF1 knockdown at the RNA level and demonstrated that reduced LEF1 expression affected the mRNA levels of SOCS1 and SOCS3, which are negative regulators of immune responses (**Supplementary Figure S2C**, **Figures 3A, B**). As suppressors of cytokine signaling, SOCS1 and SOCS3 modulate immune responses by inhibiting cytokine signaling pathways (41). To further validate the pro-inflammatory effects of LEF1 downregulation, we performed ELISA assays. The results showed a significant increase in IL-6 and TNF-α levels in the supernatants of LEF1-knockdown cells (**Figures 3C, D**).

### LEF1 deficiency in microglia promotes neuroinflammation and oxidative damage

**Figure 4:**
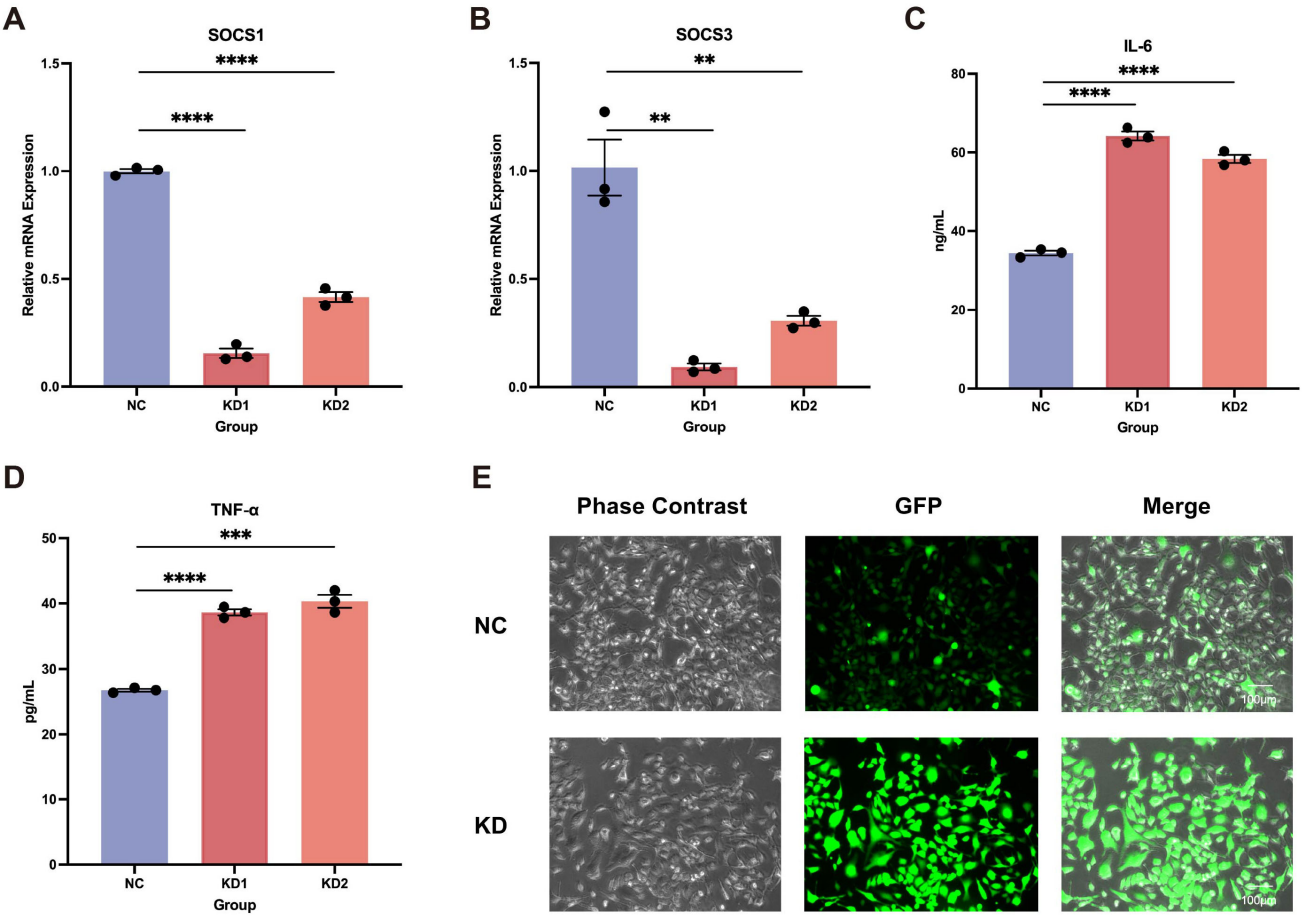
Impact of LEF1 knockdown on microglial. **(A, B)** RT-qPCR results indicate that LEF1 knockdown leads to a decrease in SOCS1 and SOCS3 expression levels. **(C, D)** ELISA analysis of cell supernatants following LEF1 knockdown revealed increased levels of IL-6 and TNF-α. **(E)** Fluorescence staining was used to assess intracellular ROS levels following LEF1 knockdown, with ROS appearing as green fluorescence. The results indicated an increase in ROS levels. Data are presented as the mean ± SEM of three independent samples, and the *P* values were analyzed using a two-tailed unpaired *t* test. **P<0.01, ***P<0.001, ****P<0.0001.

In addition to its specific expression in immune cells, particularly T cells, we found that LEF1 is also specifically expressed in microglia (**Supplementary Figure S3A**). Further analysis revealed that LEF1 expression progressively declined with age in both human brain tissues (based on the BrainSpan Atlas, which profiles gene expression across human brain development) and mouse brain microglia (GSE62420) (**Supplementary Figures S3B, C**), suggesting a potential role of LEF1 in age-related central nervous system regulation (27, 42). Given our previous analysis indicating a strong association between LEF1 and neurodegenerative diseases, as well as its loss promoting inflammatory responses, we hypothesized that LEF1 might mediate neurodegenerative disease progression by regulating neuroinflammation. Therefore, we further investigated the function of LEF1 in human microglial HMC3 cells. Consistent with our findings in Jurkat cells, LEF1 knockdown in microglial cells resulted in a reduction in SOCS1 and SOCS3 expression, accompanied by a significant increase in IL-6 and TNF-α levels in the culture supernatant (**Figures 4A-D**, **Supplementary Figure S3D**). Additionally, we evaluated whether LEF1 knockdown affects ROS levels in HMC3 cells. ROS was detected using fluorescence staining, where green fluorescence intensity positively correlates with ROS levels. The results showed that LEF1 knockdown significantly increased ROS levels in microglial cells (**Figures 4E**, **Supplementary Figure S3E**). Notably, oxidative damage is a key mechanism underlying the pathogenesis of neurodegenerative diseases (43, 44).

### DNA methylation-mediated epigenetic suppression of LEF1 during aging

**Figure 5:**
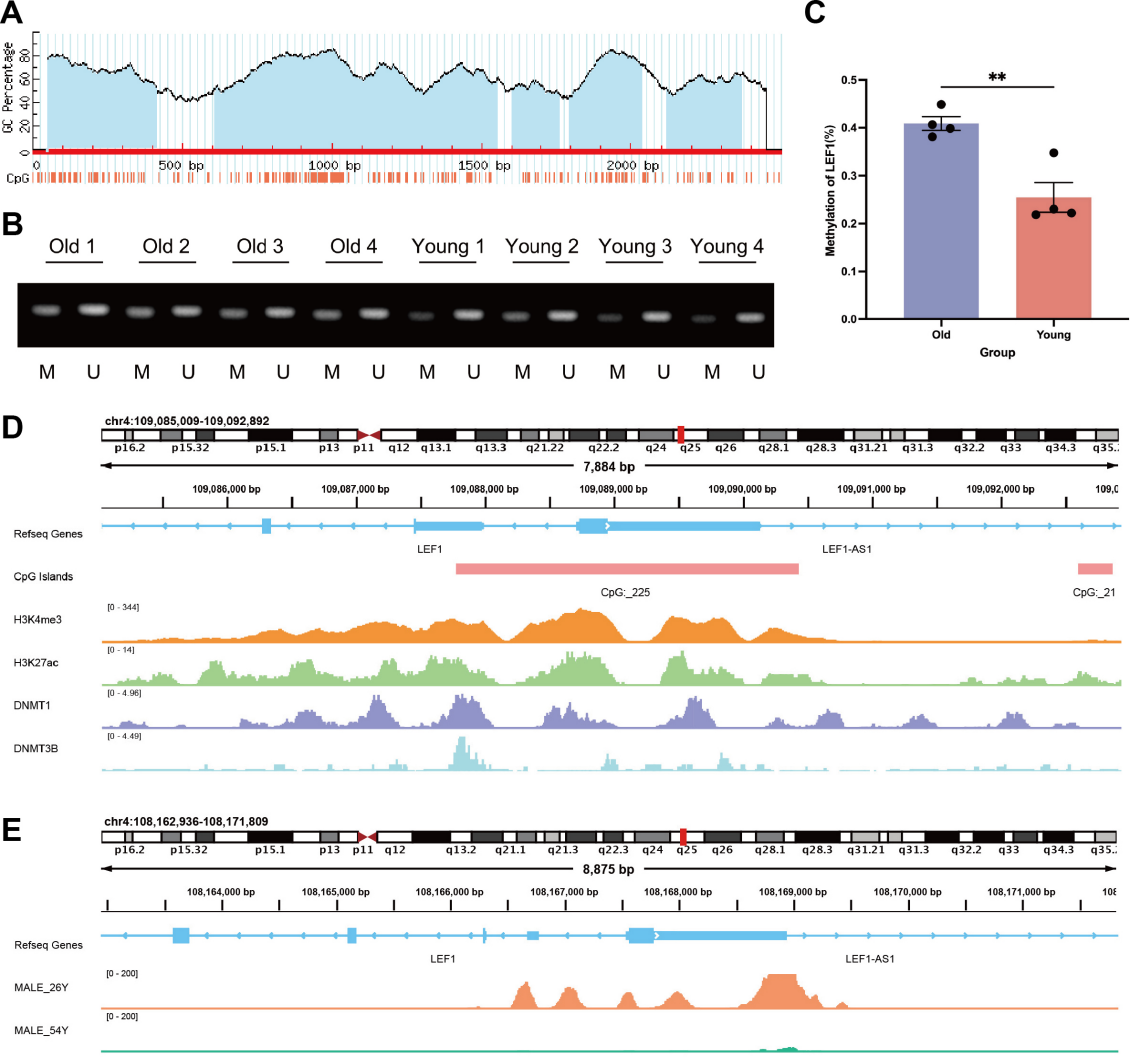
DNA methylation and chromatin accessibility changes in the LEF1 promoter region during aging. **(A)** MethPrimer software predicted the presence of CpG islands in the LEF1 promoter region. **(B)** To distinguish the methylation level of the LEF1 gene, we extracted DNA from different age groups and performed methylation-specific PCR analysis, where M represents the methylated PCR product (143 bp) and U represents the unmethylated PCR product (147 bp). **(C)** Quantitative analysis of LEF1 gene promoter methylation levels in different age groups. **(D)** Binding characteristics of different factors in the LEF1 promoter region. H3K4me3 and H3K27ac marks indicate promoter characteristics of this region, which also contains the CpG_225 site, while DNMT1 and DNMT3B binding peaks are detected in this region. **(E)** There are differences in the ATAC peak values of the LEF1 promoter region between males aged 26 and 54. As age increases, the peak values gradually decrease, indicating a corresponding reduction in chromatin accessibility. Data are presented as the mean ± SEM of three independent samples, and the *P* values were analyzed using a two-tailed unpaired *t* test. **P<0.01.

Previously, we identified methylation probes in the LEF1 promoter region that are associated with aging characteristics (**Supplementary Table S2**). We hypothesized that the decreased expression of LEF1 during aging might be related to changes in DNA methylation patterns. To test this hypothesis, we recruited 4 elderly individuals over the age of 65 and 4 young individuals between the ages of 18-25, collected their peripheral blood, and isolated PBMCs. RT-qPCR results showed a significant decrease in LEF1 expression in the elderly group (**Supplementary Figure S4**). To further investigate the DNA methylation status of the LEF1 promoter region during aging, we performed methylation-specific PCR (MSP). First, using the MethPrimer software, we identified a CpG island in the LEF1 promoter region (**Figure 5A**). The MSP results revealed a significant increase in the methylation level of CpG sites in the promoter region of LEF1 in healthy elderly individuals compared to young individuals (*P* < 0.01) (**Figures 5B, C**).

Additionally, methylation probes in regions near LEF1 have been reported to show increased methylation levels during aging (**Table 4**). ChIP-seq data from the ENCODE database further confirmed the binding signals of DNA methyltransferases DNMT1 and DNMT3B in the LEF1 promoter region, and this region includes the CpG_225 site (**Figure 5D**), suggesting that LEF1 expression may be regulated by DNA methylation. Beyond DNA methylation, we also analyzed ATAC-seq data from males of different ages. The results revealed that chromatin accessibility in the LEF1 promoter region decreased with age (**Figure 5E**). These findings collectively suggest that LEF1 expression during aging may be regulated by DNA methylation-mediated epigenetic modifications.

**Table 4:** Methylation probes associated with aging traits located in the vicinity of the LEF1 gene region.

Probe ID	Correlations	Location	Related genes (transcript: location)	CpG islands	Related traits
cg07905908	Hyper: 100%;Hypo: 0%;NR: 0%	chr4: 109034624	LEF1 (ENST00000265165: body);LEF1 (ENST00000379951: body);LEF1 (ENST00000510135: body);LEF1 (ENST00000438313: body);LEF1 (ENST00000509428: body);LEF1 (ENST00000510624: body);LEF1 (ENST00000506680: body);LEF1 (ENST00000504775: body);LEF1 (ENST00000504950: body);LEF1 (ENST00000515500: body);LEF1 (ENST00000510717: body);LEF1 (ENST00000512172: body);LEF1 (ENST00000505293: body);	Other	aging;
cg16642281	Hyper: 100%;Hypo: 0%;NR: 0%	chr4: 109034555	LEF1 (ENST00000265165: body);LEF1 (ENST00000379951: body);LEF1 (ENST00000510135: body);LEF1 (ENST00000438313: body);LEF1 (ENST00000509428: body);LEF1 (ENST00000510624: body);LEF1 (ENST00000506680: body);LEF1 (ENST00000504775: body);LEF1 (ENST00000504950: body);LEF1 (ENST00000515500: body);LEF1 (ENST00000510717: body);LEF1 (ENST00000512172: body);LEF1 (ENST00000505293: body);	Other	prostate cancer;aging;
cg06408078	Hyper: 100%;Hypo: 0%;NR: 0%	chr4: 109090828	LEF1 (ENST00000265165: promoter);LEF1 (ENST00000379951: promoter);RP11-558N14.1 (ENST00000436413: body);	Shore	aging;
cg19692648	Hyper: 100%;Hypo: 0%;NR: 0%	chr4: 109087980	LEF1 (ENST00000265165: body);LEF1 (ENST00000379951: body);LEF1 (ENST00000438313: promoter);LEF1 (ENST00000510624: promoter);LEF1 (ENST00000506680: promoter);LEF1 (ENST00000504775: promoter);LEF1 (ENST00000504950: promoter);LEF1 (ENST00000515500: promoter);LEF1 (ENST00000512172: promoter);LEF1 (ENST00000505293: promoter);RP11-558N14.1 (ENST00000436413: promoter);	Island	aging;smoking;

## Discussion

With the growing aging population, increasing attention has been directed toward understanding the mechanisms and molecular changes associated with aging. Although addressing the root causes of aging to extend human lifespan remains a significant challenge, studies have suggested that epigenetic regulation is a primary driving force of aging, offering the possibility of reversing age-related decline (15, 45, 46). In this study, we analyzed datasets from the GEO database to identify genes with altered expression during the aging process. Among these, LEF1 was found to harbor aging-associated methylation probes in its promoter region and exhibited significantly reduced expression in elderly individuals. Subsequently, we employed a range of bioinformatics approaches—including correlation analysis, differential expression analysis, PPI network analysis, and ChIP-seq, to comprehensively investigate the aberrant pathways potentially regulated by LEF1 during aging.

LEF1 belongs to the T cell factor (TCF)/LEF family of transcription factors and functions as a nuclear effector in the Wnt/β-catenin signaling pathway (47, 48). Numerous studies have reported that aberrant expression of LEF1 is associated with tumorigenesis, as well as cancer cell proliferation, migration, and invasion, and its overexpression has been linked to poor prognosis (49–53). Previous research has identified LEF1 as one of the few immune-related genes consistently downregulated with age across multiple species and tissues, suggesting a conserved and essential biological role in age-related immune regulation and various signaling pathways (54). In multiple immune cell types from both humans and mice, LEF1 has been found to act as a commonly age-associated transcriptional regulator, with isoform-specific changes in expression potentially representing a key mechanism driving cellular senescence (55). LEF1 dysregulation appears to be an intrinsic feature of the aging process, contributing to cellular dysfunction and increased susceptibility to age-related diseases.

During aging, the immune system undergoes a poorly defined process of immunosenescence, characterized by progressive immune dysfunction, chronic inflammation, and features of autoimmunity (13, 56, 57). Increased levels of inflammatory mediators are strongly associated with the development of most age-related chronic conditions, including neurodegenerative diseases and cancer (58, 59). Cognitive decline is a hallmark of human aging, and neuroinflammation appears to be a major contributor to age-related cognitive impairment (14, 60). Chronic low-grade inflammation in the periphery may promote neuroinflammatory processes in the aging brain by modulating glial cell activation and the expression of inflammatory cytokines, ultimately leading to neuronal dysfunction and the accumulation of brain tissue damage—even in cognitively intact elderly individuals (14, 61, 62). In our study, we observed a significant decrease in LEF1 expression in the peripheral blood of elderly individuals. Correlation and functional analyses indicated that LEF1 is closely associated with immune inflammation and neurodegenerative diseases. As a nuclear transcription factor, LEF1 was found, through ChIP-seq analysis, to bind target genes that are predominantly enriched in pathways related to neurodegeneration. Knockdown of LEF1 in both T-cell lines and microglial cells resulted in a marked increase in IL-6 and TNF-α levels. As central regulators of neuroinflammation, microglial cells exhibit robust IL-6 responses, with basal expression levels significantly higher than those observed in Jurkat T cells. Despite this baseline difference, both cell types showed a consistent trend of increased IL-6 expression upon LEF1 knockdown, suggesting that LEF1 exerts a relatively conserved anti-inflammatory role across distinct immune cell types.

In addition, we found that knockdown of LEF1 in microglial cells led to a significant increase in ROS levels. Microglia, often referred to as the “brain-resident macrophages,” are complex and dynamic mediators of neuroinflammation and key regulators of immune responses in neurodegenerative diseases (63, 64). Microglial activation and oxidative stress are hallmarks of neurodegeneration and are known to drive disease progression (43). Studies have suggested that oxidative stress is a defining feature of Alzheimer’s disease, characterized by lipid peroxidation, protein oxidation, and mitochondrial DNA damage (65–67). Microglia-derived ROS may further exacerbate oxidative stress associated with neurodegenerative pathology. However, whether LEF1 regulates ROS production through direct transcriptional control of oxidative enzymes or indirectly (like via modulation of inflammatory signaling pathways) remains to be clarified, and further mechanistic studies are warranted.

During aging, epigenetic mechanisms, particularly changes in DNA methylation patterns, alter the expression of aging-associated genes and represent one of the key drivers of cellular functional decline and the development of age-related diseases (7, 23). After identifying aging-associated methylation probes within the promoter region of the aging-related biomarker LEF1, we recruited healthy volunteers across different age groups to validate changes in its expression. The results showed a significant reduction in LEF1 expression in elderly individuals. Using methylation-specific PCR, we confirmed that the methylation level of the LEF1 promoter region was elevated in older adults. ChIP-seq analysis further supported this finding by revealing CpG islands and DNA methyltransferase binding peaks within the LEF1 promoter region. Moreover, ATAC-seq data demonstrated age-dependent differences in chromatin accessibility at the LEF1 promoter, with a decline in chromatin openness observed in older individuals. Although the sample size was limited, these results suggest that chromatin accessibility at the LEF1 promoter may undergo dynamic changes during aging and provide a valuable reference for future studies, though the underlying mechanisms warrant further investigation. In addition, the downregulation of LEF1 expression may also be influenced by other mechanisms, such as age-associated changes in upstream transcription factors (e.g., ERG), microRNA-mediated post-transcriptional repression, or alterations in RNA stability (e.g., involvement of natural antisense transcripts or alternative splicing) (68–72). These mechanisms were not explored in the present study and warrant further investigation in future research.

Despite these findings, our study has certain limitations that warrant consideration. First, although we validated some key findings using *in vitro* cellular models, further *in vivo* functional experiments are necessary to fully elucidate the role of LEF1 in aging and neuroinflammation. Second, while our integrative multi-omics analysis provides preliminary insights into the epigenetic regulatory mechanisms and potential downstream targets of LEF1, its direct regulatory interactions require further confirmation, such as by chromatin immunoprecipitation followed by qPCR (ChIP-qPCR). Moreover, although our findings suggest a potential involvement of LEF1 in the regulation of neurodegenerative processes, the precise signaling axis and underlying molecular mechanisms remain to be clarified.

**Figure 6:**
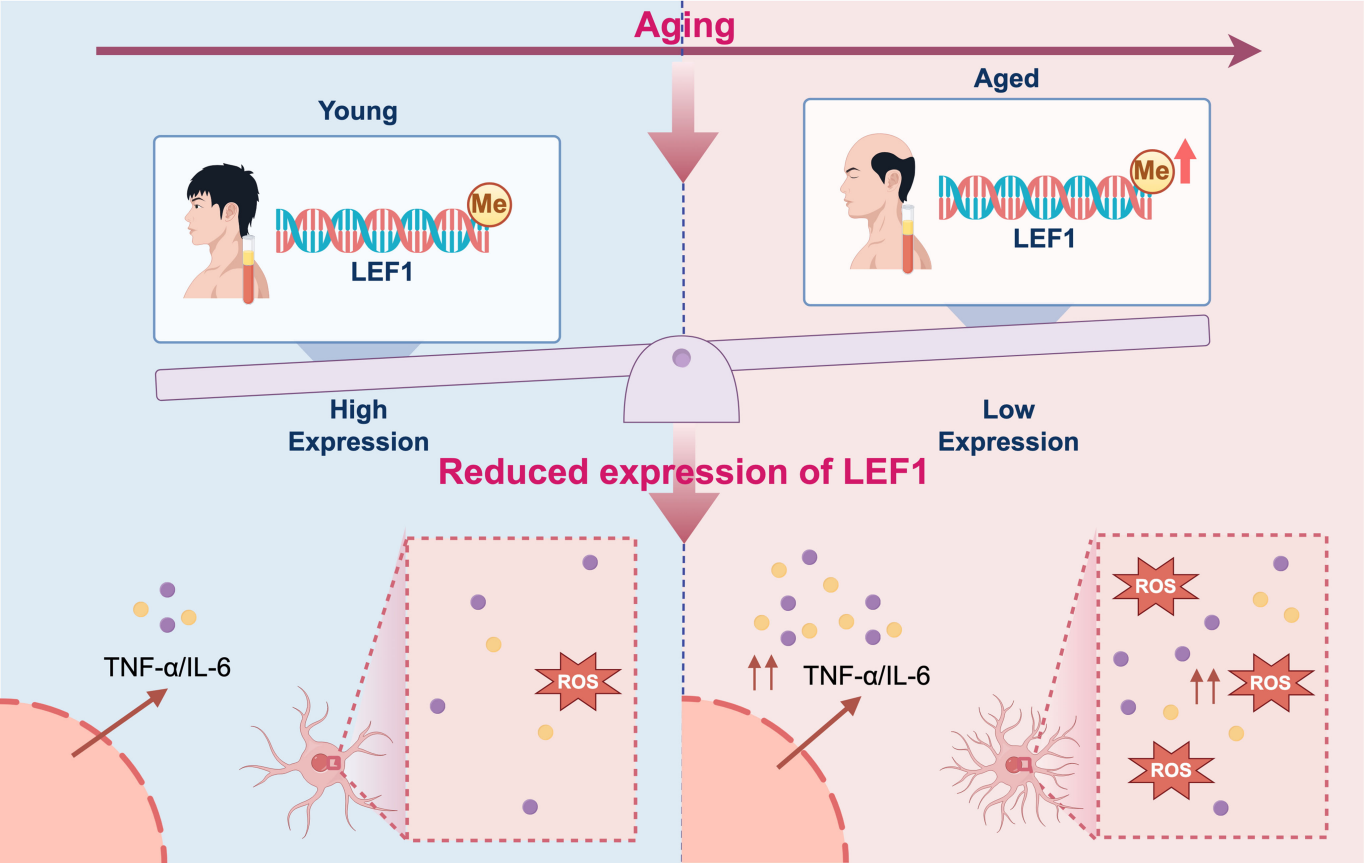
Proposed mechanistic model of inflammation driven by epigenetic repression of LEF1 during aging. During aging, DNA methylation in the promoter region of LEF1 leads to its transcriptional repression. The downregulation of LEF1 impairs its anti-inflammatory regulatory functions, resulting in elevated production of pro-inflammatory cytokines IL-6, TNF-α, and ROS levels. This epigenetically driven reduction in LEF1 expression may contribute to chronic low-grade inflammation, a hallmark of aging and age-related diseases. The figure was visualized by FigDraw.

In summary, this study reveals that LEF1 is subject to epigenetic regulation during aging, and its downregulation may contribute to enhanced inflammatory responses and oxidative stress (**Figure 6**). Our findings provide new insights and potential directions for exploring the molecular mechanisms and therapeutic targets of age-related diseases.

## Data Availability

The raw data supporting the conclusions of this article will be made available by the authors, without undue reservation.

## References

[1] Guo J Huang X Dou L Yan M Shen T Tang W. Aging and aging-related diseases: from molecular mechanisms to interventions and treatments. Signal Transduct Target Ther. (2022) 7:391. doi: 10.1038/s41392-022-01251-0, PMID: 36522308 PMC9755275

[2] Lopez-Otin C Blasco MA Partridge L Serrano M Kroemer G. Hallmarks of aging: An expanding universe. Cell. (2023) 186:243–78. doi: 10.1016/j.cell.2022.11.001, PMID: 36599349

[3] Li Y Tian X Luo J Bao T Wang S Wu X. Molecular mechanisms of aging and anti-aging strategies. Cell Commun Signal. (2024) 22:285. doi: 10.1186/s12964-024-01663-1, PMID: 38790068 PMC11118732

[4] Lopez-Otin C Blasco MA Partridge L Serrano M Kroemer G. The hallmarks of aging. Cell. (2013) 153:1194–217. doi: 10.1016/j.cell.2013.05.039, PMID: 23746838 PMC3836174

[5] la Torre A Lo Vecchio F Greco A. Epigenetic mechanisms of aging and aging-associated diseases. Cells. (2023) 12:1163. doi: 10.3390/cells12081163, PMID: 37190071 PMC10136616

[6] Montegut L Lopez-Otin C Kroemer G. Aging and cancer. Mol Cancer. (2024) 23:106. doi: 10.1186/s12943-024-02020-z, PMID: 38760832 PMC11102267

[7] Horvath S Raj K. DNA methylation-based biomarkers and the epigenetic clock theory of ageing. Nat Rev Genet. (2018) 19:371–84. doi: 10.1038/s41576-018-0004-3, PMID: 29643443

[8] Basisty N Kale A Jeon OH Kuehnemann C Payne T Rao C. A proteomic atlas of senescence-associated secretomes for aging biomarker development. PloS Biol. (2020) 18:e3000599. doi: 10.1371/journal.pbio.3000599, PMID: 31945054 PMC6964821

[9] Evans DS Young D Tanaka T Basisty N Bandinelli S Ferrucci L. Proteomic analysis of the senescence-associated secretory phenotype: GDF-15, IGFBP-2, and cystatin-C are associated with multiple aging traits. J Gerontol A Biol Sci Med Sci. (2024) 79. doi: 10.1093/gerona/glad265, PMID: 37982669 PMC10876076

[10] Wang B Han J Elisseeff JH Demaria M. The senescence-associated secretory phenotype and its physiological and pathological implications. Nat Rev Mol Cell Biol. (2024) 25:958–78. doi: 10.1038/s41580-024-00727-x, PMID: 38654098

[11] Garcia-Dominguez M. Pathological and inflammatory consequences of aging. Biomolecules. (2025) 15(3):404. doi: 10.3390/biom15030404, PMID: 40149940 PMC11939965

[12] Silva BA Farias MI Miglietta EA Leal MC Avalos JC Pitossi FJ. Understanding the role of the blood brain barrier and peripheral inflammation on behavior and pathology on ongoing confined cortical lesions. Mult Scler Relat Disord. (2022) 57:103346. doi: 10.1016/j.msard.2021.103346, PMID: 35158455

[13] Di Benedetto S Müller L. Aging, Immunity, and Neuroinflammation: The Modulatory Potential of Nutrition. In: Mahmoudi M Rezaei N, editors. Nutrition and Immunity. Springer International Publishing, Cham (2019). p. 301–22.

[14] Di Benedetto S Muller L Wenger E Duzel S Pawelec G. Contribution of neuroinflammation and immunity to brain aging and the mitigating effects of physical and cognitive interventions. Neurosci Biobehav Rev. (2017) 75:114–28. doi: 10.1016/j.neubiorev.2017.01.044, PMID: 28161508

[15] Yang JH Hayano M Griffin PT Amorim JA Bonkowski MS Apostolides JK. Loss of epigenetic information as a cause of mammalian aging. Cell. (2023) 186:305–26 e27. doi: 10.1016/j.cell.2022.12.027, PMID: 36638792 PMC10166133

[16] Pan C Zhou F Zhang L. The loss of epigenetic information: not only consequences but a cause of mammalian aging. Signal Transduct Target Ther. (2023) 8:140. doi: 10.1038/s41392-023-01412-9, PMID: 36973241 PMC10042813

[17] Booth LN Brunet A. The aging epigenome. Mol Cell. (2016) 62:728–44. doi: 10.1016/j.molcel.2016.05.013, PMID: 27259204 PMC4917370

[18] Benayoun BA Pollina EA Brunet A. Epigenetic regulation of ageing: linking environmental inputs to genomic stability. Nat Rev Mol Cell Biol. (2015) 16:593–610. doi: 10.1038/nrm4048, PMID: 26373265 PMC4736728

[19] Stubbs TM Bonder MJ Stark AK Krueger F Team BIAC von Meyenn F. Multi-tissue DNA methylation age predictor in mouse. Genome Biol. (2017) 18:68. doi: 10.1186/s13059-017-1203-5, PMID: 28399939 PMC5389178

[20] Hannum G Guinney J Zhao L Zhang L Hughes G Sadda S. Genome-wide methylation profiles reveal quantitative views of human aging rates. Mol Cell. (2013) 49:359–67. doi: 10.1016/j.molcel.2012.10.016, PMID: 23177740 PMC3780611

[21] Horvath S. DNA methylation age of human tissues and cell types. Genome Biol. (2013) 14:R115. doi: 10.1186/gb-2013-14-10-r115, PMID: 24138928 PMC4015143

[22] Jylhava J Pedersen NL Hagg S. Biological age predictors. EBioMedicine. (2017) 21:29–36. doi: 10.1016/j.ebiom.2017.03.046, PMID: 28396265 PMC5514388

[23] Unnikrishnan A Freeman WM Jackson J Wren JD Porter H Richardson A. The role of DNA methylation in epigenetics of aging. Pharmacol Ther. (2019) 195:172–85. doi: 10.1016/j.pharmthera.2018.11.001, PMID: 30419258 PMC6397707

[24] Bird A. DNA methylation patterns and epigenetic memory. Genes Dev. (2002) 16:6–21. doi: 10.1101/gad.947102, PMID: 11782440

[25] Kuparinen T Marttila S Jylhava J Tserel L Peterson P Jylha M. Cytomegalovirus (CMV)-dependent and -independent changes in the aging of the human immune system: a transcriptomic analysis. Exp Gerontol. (2013) 48:305–12. doi: 10.1016/j.exger.2012.12.010, PMID: 23291591

[26] Sirma Ekmekci S Emrence Z Abaci N Sariman M Salman B Ekmekci CG. LEF1 induces DHRS2 gene expression in human acute leukemia jurkat T-cells. Turk J Haematol. (2020) 37:226–33. doi: 10.4274/tjh.galenos.2020.2020.0144, PMID: 32586085 PMC7702649

[27] Grabert K Michoel T Karavolos MH Clohisey S Baillie JK Stevens MP. Microglial brain region-dependent diversity and selective regional sensitivities to aging. Nat Neurosci. (2016) 19:504–16. doi: 10.1038/nn.4222, PMID: 26780511 PMC4768346

[28] Ritchie ME Phipson B Wu D Hu Y Law CW Shi W. limma powers differential expression analyses for RNA-sequencing and microarray studies. Nucleic Acids Res. (2015) 43:e47. doi: 10.1093/nar/gkv007, PMID: 25605792 PMC4402510

[29] Chen H Boutros PC. VennDiagram: a package for the generation of highly-customizable Venn and Euler diagrams in R. BMC Bioinf. (2011) 12:35. doi: 10.1186/1471-2105-12-35, PMID: 21269502 PMC3041657

[30] Newman AM Liu CL Green MR Gentles AJ Feng W Xu Y. Robust enumeration of cell subsets from tissue expression profiles. Nat Methods. (2015) 12:453–7. doi: 10.1038/nmeth.3337, PMID: 25822800 PMC4739640

[31] Xie Z Bailey A Kuleshov MV Clarke DJB Evangelista JE Jenkins SL. Gene set knowledge discovery with enrichr. Curr Protoc. (2021) 1:e90. doi: 10.1002/cpz1.v1.3, PMID: 33780170 PMC8152575

[32] Szklarczyk D Kirsch R Koutrouli M Nastou K Mehryary F Hachilif R. The STRING database in 2023: protein-protein association networks and functional enrichment analyses for any sequenced genome of interest. Nucleic Acids Res. (2023) 51:D638–D46. doi: 10.1093/nar/gkac1000, PMID: 36370105 PMC9825434

[33] Shannon P Markiel A Ozier O Baliga NS Wang JT Ramage D. Cytoscape: a software environment for integrated models of biomolecular interaction networks. Genome Res. (2003) 13:2498–504. doi: 10.1101/gr.1239303, PMID: 14597658 PMC403769

[34] Xiong Z Yang F Li M Ma Y Zhao W Wang G. EWAS Open Platform: integrated data, knowledge and toolkit for epigenome-wide association study. Nucleic Acids Res. (2022) 50:D1004–D9. doi: 10.1093/nar/gkab972, PMID: 34718752 PMC8728289

[35] Luo Y Hitz BC Gabdank I Hilton JA Kagda MS Lam B. New developments on the Encyclopedia of DNA Elements (ENCODE) data portal. Nucleic Acids Res. (2020) 48:D882–D9. doi: 10.1093/nar/gkz1062, PMID: 31713622 PMC7061942

[36] Robinson JT Thorvaldsdottir H Winckler W Guttman M Lander ES Getz G. Integrative genomics viewer. Nat Biotechnol. (2011) 29:24–6. doi: 10.1038/nbt.1754, PMID: 21221095 PMC3346182

[37] Li LC Dahiya R. MethPrimer: designing primers for methylation PCRs. Bioinformatics. (2002) 18:1427–31. doi: 10.1093/bioinformatics/18.11.1427, PMID: 12424112

[38] Duclot F Kabbaj M. The role of early growth response 1 (EGR1) in brain plasticity and neuropsychiatric disorders. Front Behav Neurosci. (2017) 11:35. doi: 10.3389/fnbeh.2017.00035, PMID: 28321184 PMC5337695

[39] McNair K Spike R Guilding C Prendergast GC Stone TW Cobb SR. A role for RhoB in synaptic plasticity and the regulation of neuronal morphology. J Neurosci. (2010) 30:3508–17. doi: 10.1523/JNEUROSCI.5386-09.2010, PMID: 20203211 PMC6634083

[40] Huang ZH Feng AY Liu J Zhou L Zhou B Yu P. Inhibitor of DNA binding 2 accelerates nerve regeneration after sciatic nerve injury in mice. Neural Regener Res. (2021) 16:2542–8. doi: 10.4103/1673-5374.313054, PMID: 33907046 PMC8374553

[41] Yoshimura A Naka T Kubo M. SOCS proteins, cytokine signalling and immune regulation. Nat Rev Immunol. (2007) 7:454–65. doi: 10.1038/nri2093, PMID: 17525754

[42] Lein ES Hawrylycz MJ Ao N Ayres M Bensinger A Bernard A. Genome-wide atlas of gene expression in the adult mouse brain. Nature. (2007) 445:168–76. doi: 10.1038/nature05453, PMID: 17151600

[43] Simpson DSA Oliver PL. ROS generation in microglia: understanding oxidative stress and inflammation in neurodegenerative disease. Antioxidants (Basel). (2020) 9(8):743. doi: 10.3390/antiox9080743, PMID: 32823544 PMC7463655

[44] Yeung AWK Tzvetkov NT Georgieva MG Ognyanov IV Kordos K Jozwik A. Reactive oxygen species and their impact in neurodegenerative diseases: literature landscape analysis. Antioxid Redox Signal. (2021) 34:402–20. doi: 10.1089/ars.2019.7952, PMID: 32030995

[45] Kennedy BK Gotta M Sinclair DA Mills K McNabb DS Murthy M. Redistribution of silencing proteins from telomeres to the nucleolus is associated with extension of life span in S. cerevisiae Cell. (1997) 89:381–91. doi: 10.1016/S0092-8674(00)80219-6, PMID: 9150138

[46] Sinclair DA Mills K Guarente L. Accelerated aging and nucleolar fragmentation in yeast sgs1 mutants. Science. (1997) 277:1313–6. doi: 10.1126/science.277.5330.1313, PMID: 9271578

[47] Tan RJ Zhou D Zhou L Liu Y. Wnt/beta-catenin signaling and kidney fibrosis. Kidney Int Suppl (2011). (2014) 4:84–90. doi: 10.1038/kisup.2014.16, PMID: 26312156 PMC4536962

[48] Clevers H. Wnt/beta-catenin signaling in development and disease. Cell. (2006) 127:469–80. doi: 10.1016/j.cell.2006.10.018, PMID: 17081971

[49] Liang J Li X Li Y Wei J Daniels G Zhong X. LEF1 targeting EMT in prostate cancer invasion is mediated by miR-181a. Am J Cancer Res. (2015) 5:1124–32., PMID: 26045991 PMC4449440

[50] Zirkel A Lederer M Stohr N Pazaitis N Huttelmaier S. IGF2BP1 promotes mesenchymal cell properties and migration of tumor-derived cells by enhancing the expression of LEF1 and SNAI2 (SLUG). Nucleic Acids Res. (2013) 41:6618–36. doi: 10.1093/nar/gkt410, PMID: 23677615 PMC3711427

[51] Kobayashi W Ozawa M. The transcription factor LEF-1 induces an epithelial-mesenchymal transition in MDCK cells independent of beta-catenin. Biochem Biophys Res Commun. (2013) 442:133–8. doi: 10.1016/j.bbrc.2013.11.031, PMID: 24269234

[52] Erdfelder F Hertweck M Filipovich A Uhrmacher S Kreuzer KA. High lymphoid enhancer-binding factor-1 expression is associated with disease progression and poor prognosis in chronic lymphocytic leukemia. Hematol Rep. (2010) 2:e3. doi: 10.4081/hr.2010.e3, PMID: 22184516 PMC3222268

[53] Eskandari E Mahjoubi F Motalebzadeh J. An integrated study on TFs and miRNAs in colorectal cancer metastasis and evaluation of three co-regulated candidate genes as prognostic markers. Gene. (2018) 679:150–9. doi: 10.1016/j.gene.2018.09.003, PMID: 30193961

[54] Barth E Srivastava A Stojiljkovic M Frahm C Axer H Witte OW. Conserved aging-related signatures of senescence and inflammation in different tissues and species. Aging (Albany NY). (2019) 11:8556–72. doi: 10.18632/aging.102345, PMID: 31606727 PMC6814591

[55] Jia M Sayed K Kapetanaki MG Dion W Rosas L Irfan S. LEF1 isoforms regulate cellular senescence and aging. Aging Cell. (2023) 22:e14024. doi: 10.1111/acel.v22.12 37961030 PMC10726832

[56] Goronzy JJ Li G Yang Z Weyand CM. The janus head of T cell aging - autoimmunity and immunodeficiency. Front Immunol. (2013) 4:131. doi: 10.3389/fimmu.2013.00131, PMID: 23761790 PMC3671290

[57] Gupta S Agrawal A. Inflammation & autoimmunity in human ageing: dendritic cells take a center stage. Indian J Med Res. (2013) 138:711–6. PMC3928701 24434323

[58] Ferrucci L Fabbri E. Inflammageing: chronic inflammation in ageing, cardiovascular disease, and frailty. Nat Rev Cardiol. (2018) 15:505–22. doi: 10.1038/s41569-018-0064-2, PMID: 30065258 PMC6146930

[59] Walker KA Basisty N Wilson DM 3rd Ferrucci L. Connecting aging biology and inflammation in the omics era. J Clin Invest. (2022) 132(14). doi: 10.1172/JCI158448, PMID: 35838044 PMC9282936

[60] Ownby RL. Neuroinflammation and cognitive aging. Curr Psychiatry Rep. (2010) 12:39–45. doi: 10.1007/s11920-009-0082-1, PMID: 20425309

[61] Giunta B Fernandez F Nikolic WV Obregon D Rrapo E Town T. Inflammaging as a prodrome to Alzheimer's disease. J Neuroinflammation. (2008) 5:51. doi: 10.1186/1742-2094-5-51, PMID: 19014446 PMC2615427

[62] von Bernhardi R Tichauer JE Eugenin J. Aging-dependent changes of microglial cells and their relevance for neurodegenerative disorders. J Neurochem. (2010) 112:1099–114. doi: 10.1111/j.1471-4159.2009.06537.x, PMID: 20002526

[63] Sankowski R Bottcher C Masuda T Geirsdottir L Sagar Sindram E. Mapping microglia states in the human brain through the integration of high-dimensional techniques. Nat Neurosci. (2019) 22:2098–110. doi: 10.1038/s41593-019-0532-y, PMID: 31740814

[64] Deczkowska A Keren-Shaul H Weiner A Colonna M Schwartz M Amit I. Disease-associated microglia: A universal immune sensor of neurodegeneration. Cell. (2018) 173:1073–81. doi: 10.1016/j.cell.2018.05.003, PMID: 29775591

[65] Pratico D Clark CM Lee VM Trojanowski JQ Rokach J FitzGerald GA. Increased 8,12-iso-iPF2alpha-VI in Alzheimer's disease: correlation of a noninvasive index of lipid peroxidation with disease severity. Ann Neurol. (2000) 48:809–12. doi: 10.1002/1531-8249(200011)48:5<809::aid-ana19>3.0.co;2-9, PMID: 11079549

[66] Smith CD Carney JM Starke-Reed PE Oliver CN Stadtman ER Floyd RA. Excess brain protein oxidation and enzyme dysfunction in normal aging and in Alzheimer disease. Proc Natl Acad Sci U S A. (1991) 88:10540–3. doi: 10.1073/pnas.88.23.10540, PMID: 1683703 PMC52964

[67] Mecocci P MacGarvey U Beal MF. Oxidative damage to mitochondrial DNA is increased in Alzheimer's disease. Ann Neurol. (1994) 36:747–51. doi: 10.1002/ana.410360510, PMID: 7979220

[68] Wu L Zhao JC Kim J Jin HJ Wang CY Yu J. ERG is a critical regulator of Wnt/LEF1 signaling in prostate cancer. Cancer Res. (2013) 73:6068–79. doi: 10.1158/0008-5472.CAN-13-0882, PMID: 23913826 PMC3790861

[69] Liu Y Yan W Zhang W Chen L You G Bao Z. MiR-218 reverses high invasiveness of glioblastoma cells by targeting the oncogenic transcription factor LEF1. Oncol Rep. (2012) 28:1013–21. doi: 10.3892/or.2012.1902, PMID: 22766851

[70] Rodriguez-Ubreva J Ciudad L van Oevelen C Parra M Graf T Ballestar E. C/EBPa-mediated activation of microRNAs 34a and 223 inhibits Lef1 expression to achieve efficient reprogramming into macrophages. Mol Cell Biol. (2014) 34:1145–57. doi: 10.1128/MCB.01487-13, PMID: 24421386 PMC3958044

[71] Beltran M Aparicio-Prat E Mazzolini R Millanes-Romero A Masso P Jenner RG. Splicing of a non-coding antisense transcript controls LEF1 gene expression. Nucleic Acids Res. (2015) 43:5785–97. doi: 10.1093/nar/gkv502, PMID: 25990740 PMC4499130

[72] Santiago L Daniels G Wang D Deng FM Lee P. Wnt signaling pathway protein LEF1 in cancer, as a biomarker for prognosis and a target for treatment. Am J Cancer Res. (2017) 7:1389–406., PMID: 28670499 PMC5489786

