# Supplementary material for: Aging-associated DNA methylation of LEF1 modulates inflammation and neurodegenerative pathways
Source: Front Immunol. 2025 Aug 21;16:1656442. doi: 10.3389/fimmu.2025.1656442 (PMC12408614; doi:10.3389/fimmu.2025.1656442)
Supplement: Supplementary file 1 [file DataSheet1.docx]

**Figure S1. LEF1 Binds to Target Genes Associated with Neurodegenerative Diseases.**


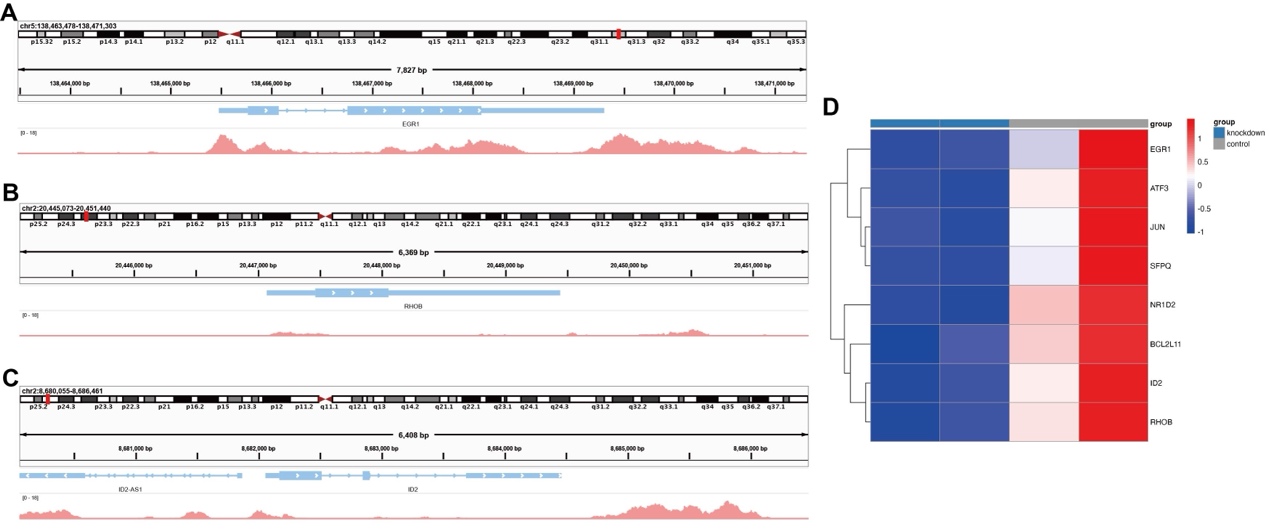


Figure S1. (A-C). Neurodegeneration-Related Genes EGR1, RHOB, and ID2 as LEF1-Associated Target Genes. (D). Downregulation of Genes in Neurodegenerative Pathways Following LEF1 Knockdown.

**Figure S2. LEF1 Expression in Various Cell Types and Confirmation of LEF1 Knockdown in Jurkat Cells.**


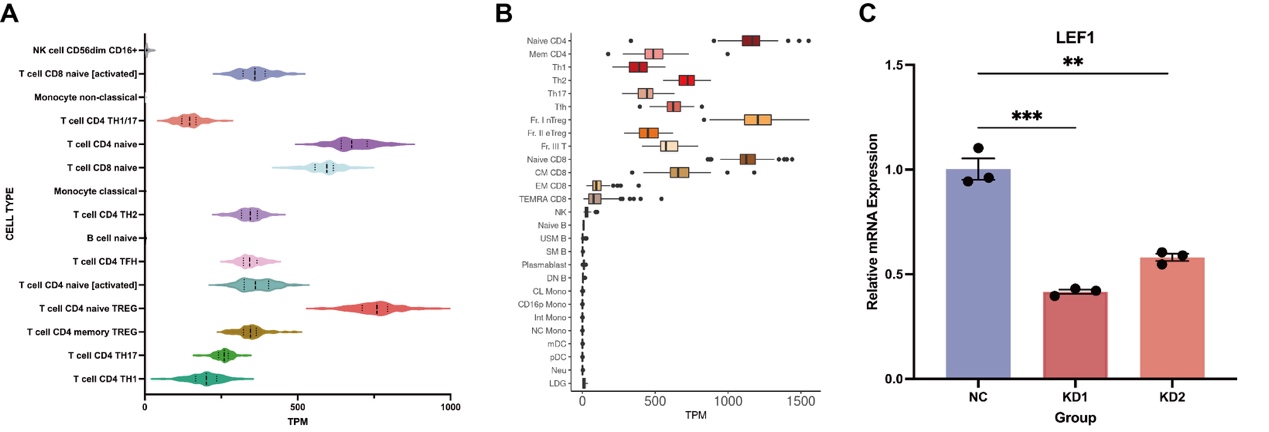


Figure S2. (A) and (B). Through the DICE and Immunexut databases, we found that LEF1 expression levels are highest in T cells compared to other cell types. (C). We confirmed LEF1 knockdown in Jurkat cells through RT-qPCR experiments. Data are presented as the mean ± SEM of three independent samples, and the *P* values were analyzed using a two-tailed unpaired *t* test. **P*<0.05, ***P*<0.01, ****P*<0.001.

**Figure S3. Expression levels of LEF1 in different cell types and its knockdown effects in HMC3 cells.**

**
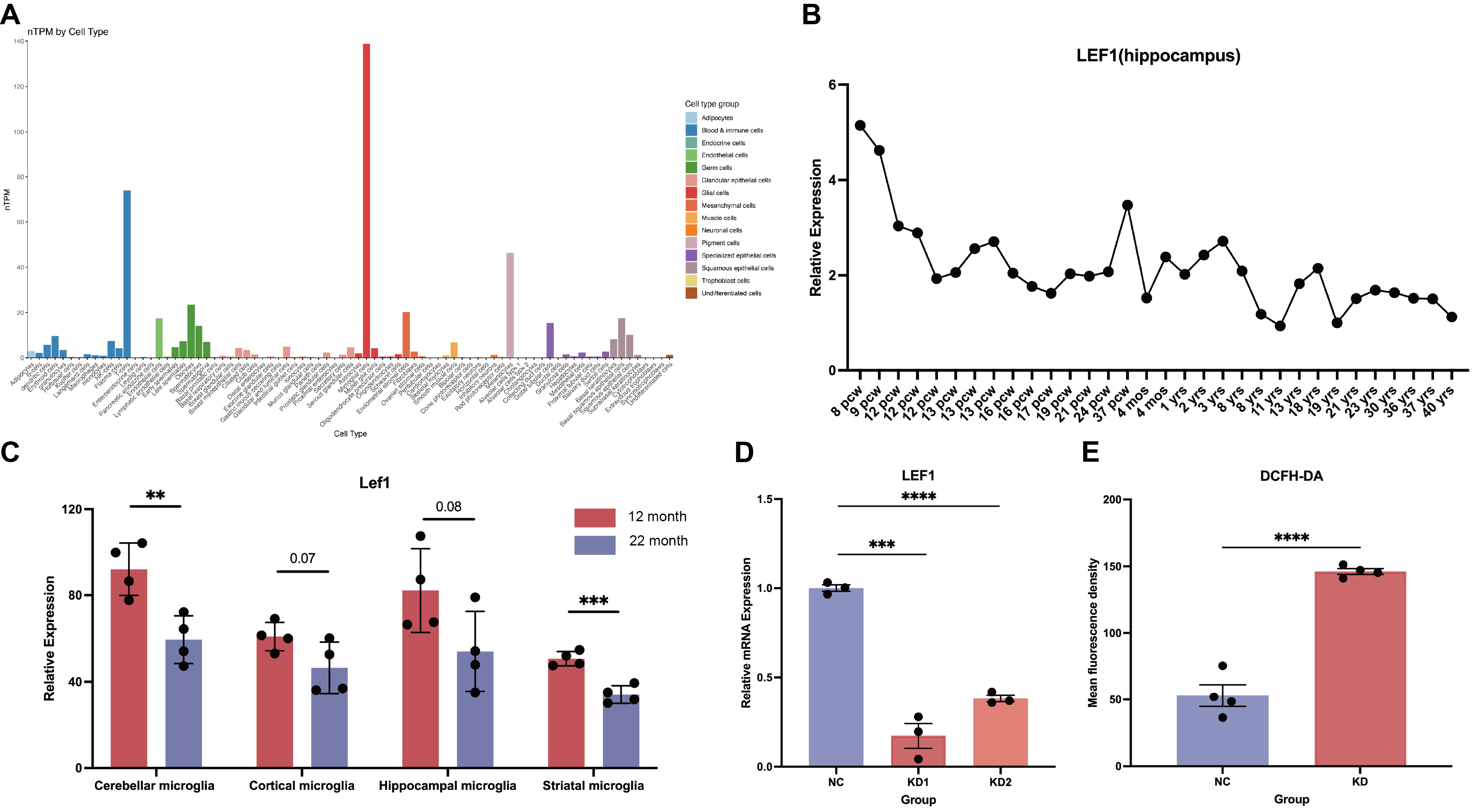
**

Figure S3. (A). LEF1 expression levels across different cell types (from The Human Protein Atlas database), with higher expression observed in microglia, T cells, and melanocytes. (B). LEF1 expression across human brain development stages based on the BrainSpan Atlas dataset. (C). LEF1 expression in mouse brain microglia from young and aged mice. (D). RT-qPCR results confirm the successful knockdown of LEF1 in HMC3 cells. (E). Quantification of intracellular ROS levels following LEF1 knockdown in microglial cells. ImageJ was used to analyze fluorescence intensity from ROS staining. Data are presented as the mean ± SEM of three independent samples, and the *P* values were analyzed using a two-tailed unpaired *t* test. **P*<0.05, ***P*<0.01, ****P*<0.001, *****P*<0.0001.

**Figure S4. LEF1 expression is significantly decreased in PBMCs of elderly individuals.**


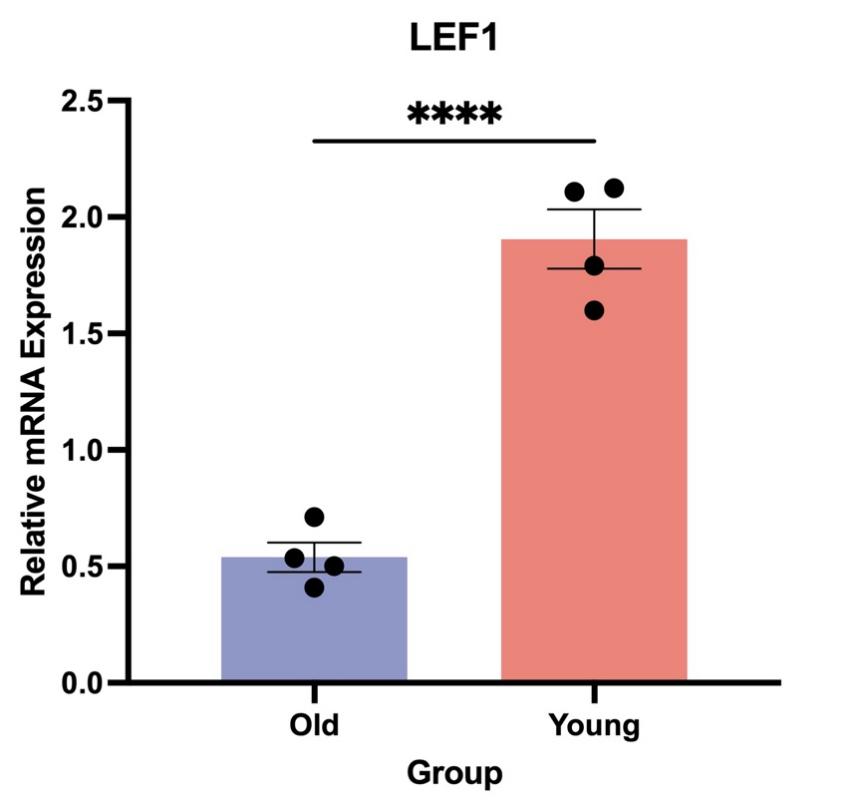


Figure S4. RT-qPCR results confirmed that LEF1 expression is significantly reduced in PBMCs of elderly individuals, which is consistent with the findings from the first part of the study. Data are presented as the mean ± SEM of three independent samples, and the *P* values were analyzed using a two-tailed unpaired *t* test. **P*<0.05, ***P*<0.01, ****P*<0.001, *****P*<0.0001.
